# Effect of Community Support on the Implementation of Primary Health Care-Based Measurement of Alcohol Consumption

**DOI:** 10.1007/s11121-021-01329-1

**Published:** 2022-01-15

**Authors:** Adriana Solovei, Eva Jané-Llopis, Liesbeth Mercken, Inés Bustamante, Daša Kokole, Juliana Mejía-Trujillo, Perla Sonia Medina Aguilar, Guillermina Natera Rey, Amy O’Donnell, Marina Piazza, Christiane Sybille Schmidt, Peter Anderson, Hein de Vries

**Affiliations:** 1 Department of Health Promotion, CAPHRI Care and Public Health Research Institute, Maastricht University, Maastricht, the Netherlands; 2 Univ. Ramon Llull, ESADE, Barcelona, Spain; 3 Institute for Mental Health Policy Research, CAMH, 33 Russell Street, Toronto, Canada; 4 School of Public Health and Administration, Universidad Peruana Cayetano Heredia, Lima, Peru; 5 Corporación Nuevos Rumbos, Bogotá, Colombia; 6 Instituto Nacional de Psiquiatría Ramón de La Fuente Muñiz, Ciudad de México, Mexico; 7 Population Health Sciences Institute, Newcastle University, Baddiley-Clark Building, Richardson Road, Newcastle upon Tyne, NE2 4AX UK; 8 Center for Interdisciplinary Addiction Research (ZIS), Department of Psychiatry and Psychotherapy, University Medical Center Hamburg-Eppendorf, Hamburg, Germany; 9 Department of Health Psychology, Faculty of Psychology, Open University, Heerlen, The Netherlands

**Keywords:** Alcohol measurement, Brief alcohol advice, Community support, Alcohol prevention, Primary health care

## Abstract

Alcohol measurement delivered by health care providers in primary health care settings is an efficacious and cost-effective intervention to reduce alcohol consumption among patients. However, this intervention is not yet routinely implemented in practice. Community support has been recommended as a strategy to stimulate the delivery of alcohol measurement by health care providers, yet evidence on the effectiveness of community support in this regard is scarce. The current study used a pre-post quasi-experimental design in order to investigate the effect of community support in three Latin American municipalities in Colombia, Mexico, and Peru on health care providers’ rates of measuring alcohol consumption in their patients. The analysis is based on the first 5 months of implementation. Moreover, the study explored possible mechanisms underlying the effects of community support, through health care providers’ awareness of support, as well as their attitudes, subjective norms, self-efficacy, and subsequent intention toward delivering the intervention. An ANOVA test indicated that community support had a significant effect on health care providers’ rates of measuring alcohol consumption in their patients (*F* (1, 259) = 4.56, *p* = 0.034, *η*_p_^2^ = 0.018). Moreover, a path analysis showed that community support had a significant indirect positive effect on providers’ self-efficacy to deliver the intervention (*b* = 0.07, *p* = 0.008), which was mediated through awareness of support. Specifically, provision of community support resulted in a higher awareness of support among health care providers (*b* = 0.31, *p* < 0.001), which then led to higher self-efficacy to deliver brief alcohol advice (*b* = 0.23, *p* = 0.010). Results indicate that adoption of an alcohol measurement intervention by health care providers may be aided by community support, by directly impacting the rates of alcohol measurement sessions, and by increasing providers’ self-efficacy to deliver this intervention, through increased awareness of support. Trial Registration ID: NCT03524599; Registered 15 May 2018; https://clinicaltrials.gov/ct2/show/NCT03524599

## Introduction

Worldwide about three million deaths are caused by alcohol every year, making alcohol consumption one of the leading preventable risk factors for physical, mental, and social harms. Alcohol is causally linked with over 200 diseases, such as cardiovascular diseases, liver cirrhosis, and various cancers (Shield et al., 2020). As in the case of smoking, alcohol not only affects the health and well-being of the individual drinker but also impacts adversely on their families, communities, and society as a whole, e.g. through increased interpersonal violence, traffic accidents, injuries, or productivity loss (WHO, 2019). Notably, one of the nine targets in the NCD global monitoring framework is a 10% relative reduction in harmful alcohol use by 2025 in comparison with 2010 (WHO, 2013).

In 2018, the World Health Organization (WHO) launched the SAFER alcohol control initiative, which entails five cost-effective strategies to combat harmful alcohol use (WHO, 2018). One of these strategies is the facilitation of patients’ access to alcohol measurement, meaning that health professionals should be actively involved in detecting and managing patterns of alcohol use in their patients. A recommended setting for this strategy is the primary health care (PHC), where the patient’s alcohol consumption can be measured by a PHC provider (e.g. physician, nurse, hereafter: provider) during a regular consultation (Anderson, 1996). However, in spite of consistent empirical evidence showing that this programme is efficacious (Kaner et al., 2018; O’Donnell et al., 2013; Platt et al., 2016) and cost-effective (Anderson et al., 2009; Solberg et al., 2008), alcohol measurement is still not widely implemented in practice (Abidi et al., 2016; Johnson et al., 2010). An important barrier encountered by providers in adopting and delivering this intervention is the (perceived) lack of support in this regard, e.g. from their managers, colleagues, and from their patients (Kokole et al., 2021; Nilsen, 2010; O’Donnell et al., 2018). A strategy repeatedly recommended to overcome this barrier is the provision of supportive actions, i.e. activities aimed at enhancing the environment in which providers must deliver the intervention (Anderson et al., 1986; Shaw et al., 1970; WHO, 2006). However, to date, few studies have explored the impact of supportive actions in this context (for example, see Anderson et al., 2017; Kaner et al., 1999). Kaner and colleagues (1999) found in their UK-based study that supportive actions (operationalized as fortnightly telephone calls to providers) had a positive impact on the delivery of alcohol measurement in a PHC setting, over and above training. Anderson and colleagues (2017) also found positive results of supportive actions (operationalized as telephone calls, as well), in a European multi-country study. However, in their study, the effect of the supportive actions could not be disentangled from that of the training.

Barker and colleagues (2015) offer an evidence-based model for increasing support in the health field, synthesizing ten areas of supportive actions deemed essential for the successful adoption, maintenance, and scale-up of a health intervention. The first five of these areas of supportive actions focus on the adoption of a health intervention (hereafter: adoption mechanisms); the other five areas focus on the maintenance of the intervention (hereafter: support systems). As such, Barker’s model aligns with previous theories and frameworks that highlight the importance of both these aspects in the sustained implementation of health interventions (Rogers, 2010). According to Barker and colleagues, the adoption mechanisms should focus on (1) positive characteristics of the intervention (e.g. effectiveness, simplicity, and congruity with the existing organizational culture), (2) involvement of leadership (e.g. in raising awareness or in the broad adoption of the intervention), (3) communication (e.g. interpersonal or mediatic messages demonstrating the value of the intervention to the leadership and implementers), (4) policy (e.g. regulatory or administrative policies that foster the adoption of the intervention), and (5) culture of urgency and persistence (e.g. ensuring that the intervention responds to an existing need and/or solves a problem). Support systems should focus on (1) human capability for scale-up (e.g. delivering sufficient training, share stories of success and challenge), (2) infrastructure for scale-up (e.g. considering whether new tools, communication systems, and key personnel are needed), (3) data collection and reporting systems (e.g. tracking implementation data and providing performance feedback), (4) learning systems (e.g. mechanisms and platforms for sharing knowledge, tools, ideas, and experiences among the implementers), and (5) design for sustainability (e.g. if needed, adapting the intervention so that it can be maintained after the end of the project).

In the international SCALA study, we drew on the model developed by Barker and colleagues (2015) to design and evaluate the impact of supportive actions developed together with local community stakeholders (henceforth: community support) on alcohol measurement in a PHC setting, in three Latin American countries (Jané-Llopis et al., 2020). Interim results are reported elsewhere (Anderson et al., 2021) and show that when analysing changes at the level of the PHC centres (PHCCs), no effects were found of community support on provision of the intervention. A possible reason for the lack of effects was the shorter implementation time of the SCALA project than initially planned (5 months vs. 18 months), due to COVID-19 restrictions. As community support is generally expected to have a cumulative effect over time, it may therefore be premature to conclude that it does not lead to the increased implementation of alcohol measurement and brief advice over time. However, another reason for the lack of observed effects could be that the unit of analysis in the study of Anderson et al. (2021) was PHCCs as a whole; meaning, we were unable to detect differences among providers working in the same PHCC. This is potentially of value because providers working in the same PHCC may perceive the community support differently, based on individual differences and socio-cognitive characteristics (Jacobs et al., 2015; Kelly et al., 2017). In the current study, we use data from the SCALA study to explore the impact of community support on the delivery of alcohol measurement but we changed the unit of analysis from the PHCC level to the provider level, thereby focusing on individual provider performance rather than PHCCs.

Additionally, it is worth exploring the effects that community support can have on socio-cognitive predictors of the desired behaviour. An increased understanding of not only whether but also how community support may influence behaviour is crucial for the further development and adaptation of effective community support. A robust theoretical framework that can be used to test the effects of community support on health behaviours and/or adoption and implementation of a health intervention is the theory of planned behaviour (TPB) (Ajzen, 1991; McDermott et al., 2015). This theory proposes that (health) behaviour is largely predicted by behavioural intention (i.e. a person’s conscious plan or decision to exert effort to engage in the behaviour), which at its turn is explained by three socio-cognitive factors: (1) attitude (i.e. the degree to which a person has a favourable or unfavourable evaluation of the behaviour of interest), (2) subjective norms (i.e. the belief about whether most people around the person approve or disapprove of his/her behaviour), and (3) perceived behavioural control, also widely known as self-efficacy (i.e. perception of the ease or difficulty of performing the behaviour of interest; hereafter: self-efficacy).

Community support can influence the attitudes towards the behaviour by highlighting the benefits and superiority of the intervention through personal, interpersonal, or mediated communication (Cialdini et al., 1981; Southwell & Yzer, 2007). Subjective norms can be influenced by community support through the involvement of leaders, managers, and/or peers as message sources, thereby promoting and popularizing widespread support for the intervention (Aarons et al., 2018). Self-efficacy can be influenced by community support through messages that particularly address the person’s confidence that he/she can perform the behaviour or by giving performance feedback, which then translates into increased confidence to (continue to) perform the behaviour (Ellen et al., 1991).

Furthermore, other more elaborate theoretical models suggest that the effect of (health) persuasion efforts, including community support, on a person’s socio-cognitive beliefs (e.g. attitude, subjective norms, self-efficacy) is mediated through the person’s awareness of these activities (De Vries, 2017; McGuire, 1989). In other words, in order for a person to change his/her beliefs and subsequent intention regarding a behaviour, as a result of being exposed to community support, the person needs to be aware that he/she was given support.

The aim of the current study is to explore whether delivery of community support has an effect on increasing alcohol measurement rates delivered by providers in primary health care settings, as well as what are possible mechanisms underlying such an effect. To account for possible confounding effects of training (which was given to a part of the participating providers), only those providers who received training (standard and/or more intensive) were included in the current analyses, as explained in more detail below. The study puts the following hypotheses forward:

- H1: Provision of community support, over and above standard training, leads to increased rates of alcohol measurement sessions delivered by PHC providers.
- H2(a–d): Provision of community support, over and above standard or more intensive training, leads to (a) increased awareness of support by PHC providers, which consequently leads to more positive or stronger: (b) attitudes; (c) subjective norms; (d) self-efficacy, subsequently resulting in a higher (e) intention to deliver alcohol measurements to their patients.

## Methods

### Study Design, Participants, and Procedure

The current study is part of the larger quasi-experimental SCALA study (Jané-Llopis et al., 2020), which tests the effectiveness of several strategies to improve the implementation of an alcohol measurement programme in three Latin American countries: Colombia, Mexico, and Peru. Specifically, in each of the three countries, two municipalities are compared: one intervention municipality (in which community support was provided) and one control municipality. The municipalities were selected by the local researchers and, in each country, were comparable in terms of socio-demographic characteristics, size, and geographical location. Randomized selection of the municipal areas was not possible because of the need to obtain approval of participation from the respective municipal authorities.

**Fig. 1:**
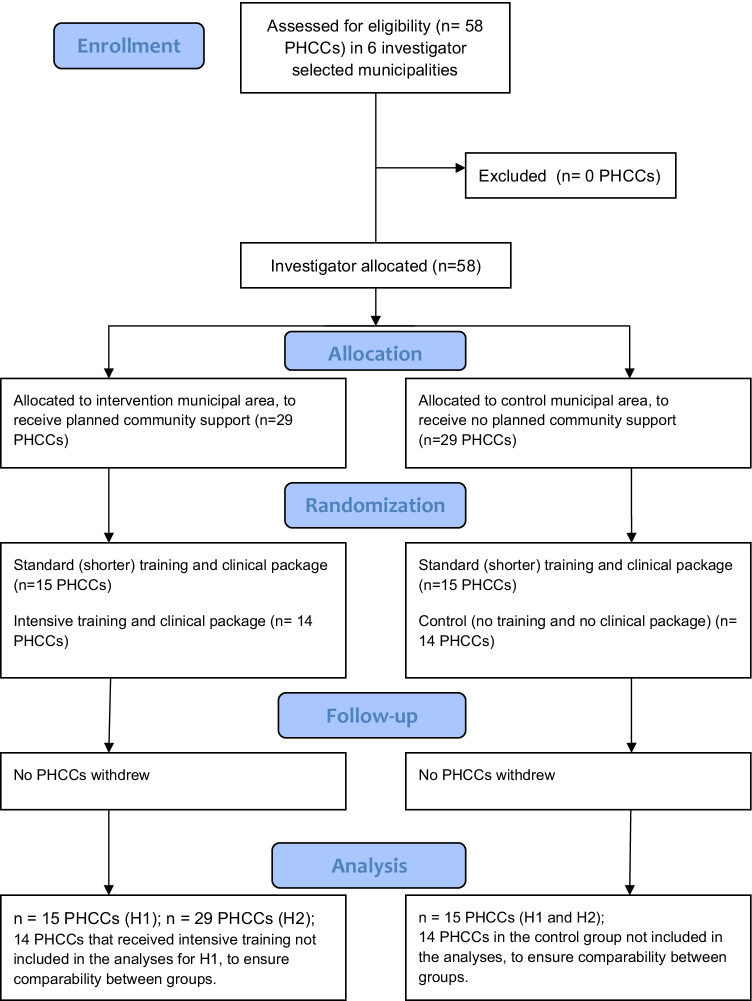
SCALA study flow based on the analyses in the current study

Within the three control municipalities, which did not receive community support, a total of 14 PHCCs were randomly allocated to a no-training condition (arm 1), and 15 PHCCs to receive standard training to implement a standard clinical package (arm 2). Within the three intervention municipalities, in which community support was provided, a total of 15 PHCCs were randomly allocated to receive standard training to implement a standard clinical package (arm 3), and 14 PHCCs to receive more intense training to implement a more intense clinical package (arm 4). Randomization was done using a random number generator in Excel. A study flow of the SCALA study, adapted for analyses in the current paper, is shown in Fig. 1.

For testing hypothesis 1, providers participating in study arms 2 and 3 were included, to ensure optimal comparability among the groups. In total, in these arms, 291 providers completed the baseline measurements and recorded the consultations in which they delivered alcohol measurement, on tally sheets, throughout the 5-month implementation period.

For testing hypothesis 2, which involves longitudinal analyses, all providers in the intervention municipality were included in the analyses (so also those in arm 4). In total, in these arms, 139 providers completed the follow-up questionnaire before data collection had to be stopped at month 5 of implementation due to the COVID-19 lockdown in the participating countries.

### Intervention

**Fig. 2:**
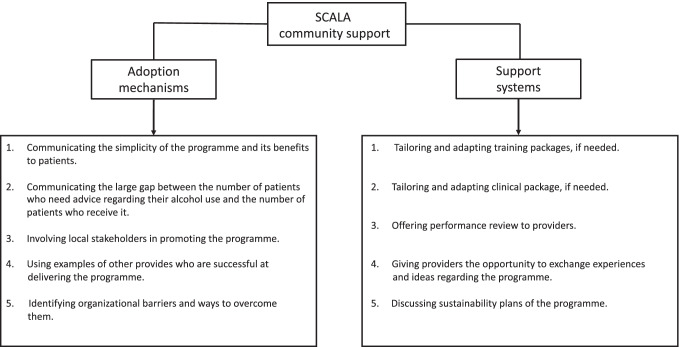
SCALA community support implemented in the first 5 months of implementation

SCALA community support was operationalized as a package of activities, planned in each of the three intervention municipalities (Solovei et al., 2021). The first phase of the community support (Fig. 2) — which is the focus of the current study — was implemented during the set-up phase (approximately 2 months) and the first five implementation months. The community support activities were developed locally, with input from and in collaboration with local stakeholders, project champions (i.e. persons who advocate the implementation of the new intervention and generate support for its adoption), and public health experts involved in the project. Moreover, in each intervention municipality, a community advisory board was formed, which held several meetings in the set-up phase of the project, to provide input for, among others, the development of plans for community support.

The implemented community support activities (Table 1) were comparable in the three implementation municipalities and included five adoption mechanisms and five support systems, based on the abovementioned recommendations by Barker and colleagues (2015). SCALA adoption mechanisms were as follows: (1) communicating to providers and representatives of the PHCCs the simplicity of the programme and its benefits to patients; (2) communicating to providers the large gap between the number of patients who need advice regarding their alcohol use and the number of patients who actually receive it; (3) involving local stakeholders in promoting alcohol measurement; (4) using examples of other provides who are successful at delivering alcohol measurement; and (5) identifying organizational barriers and ways to overcome them. SCALA support systems were as follows: (1) tailoring and adapting training packages, if needed; (2) tailoring and adapting clinical package, if needed; (3) offering performance review to providers; (4) giving providers the opportunity to exchange experiences and ideas regarding the programme; and (5) discussing sustainability plans of the programme. Additionally, a communication campaign was planned and prepared in each intervention municipality which, however, could not be fully implemented due to restrictions related to the COVID-19 pandemic.

**Table 1:** Community support activities implemented in the first five months of implementation

Community support activities	Colombia	Mexico	Peru
Adoption mechanisms	1. The benefits for patients and simplicity of the intervention were emphasized in face-to-face meetings with PHCC managers and providers. 2. In implementation month 3, in face-to-face meetings with providers, the number of patients whose alcohol consumption was measured and was communicated to providers. 3. A local university became engaged in the project and provided input on adaptations of the intervention. 4. In implementation month 3, in a face-to-face meetings with providers, the highest screening rates per PHCC were highlighted. 5. Organizational issues are monitored through discussions with PHCC, no substantial issues have been identified.	1. The benefits for patients and simplicity of the intervention were emphasized in face-to face meetings with PHCC managers and providers. 2. In face-to-face meetings with providers, the large number of patients that can benefit if screening and brief advice are implemented in the PHCC was reaffirmed. 3. A poster presentation held at an Annual Research Meeting of the National Institute of Psychiatry; a presentation about the role of alcohol screening was held on the National Day against harmful use of alcoholic beverages 2019. 4. Informing PHCCs about the percentage of screenings carried out by each PHCC, on a monthly basis. 5. Organizational issues were monitored through discussions with PHCCs, no substantial issues were identified.	1. Collaboration with the Mental Health Program of the Ministry of Health, in order to promote the adoption of the programme in the implementation municipality. 2. The large number of patients who benefit from the project is communicated to providers, focusing on three subgroups with higher alcohol risk in the intervention municipality: (a) persons in treatment of tuberculosis, (b) persons at risk of sexual transmitted diseases, (c) persons in violent families. 3. In order to engage the municipality, 35 community promoters have been trained in methods for working in alcohol prevention. 4. Lists were created for each PHCC using WhatsApp to promote the identification of champions. 5. Organizational issues are monitored through discussions with PHCCs; one issue identified is that providers seem very busy.
Support systems	1. Training packages were slightly shortened, in order to fit into the PHCCs’ schedules and rules of attendance of providers. 2. One formal meeting was organized in the first 2 months of implementation to identify difficulties regarding the brief intervention and the care pathway. It was identified that providers still needed support to get used to the exact pathway. In response, three short support videos were created, about how to fill in the tally sheets, how to mark the boxes, and what is the needed material to be delivered for each case. 3. Meetings for feedback with providers were held every 2 months, in which the screening rates are communicated. Recognitions in the form of symbolic incentives ($5 vouchers) were given to the 8–9 providers with the highest measurement rates. 4. Informal exchange of experiences among participating providers. 5. Mentions of the programmes’ potential sustainability during meetings with PHCC managers and providers.	1. Materials and activities of the training sessions (i.e. role playing, presentations and analysis of the videos) were adjusted to the needs of each PHCC. 2. Face to face meetings with providers, during which they agreed that no additional tailoring was needed. 3. Reporting each month to PHCCs’ the number of screenings; informing the PHCCs every three months on the progress of the global project. Recognitions in the form of certificates were given to the PHCC and the most outstanding suppliers each quarter. 4. Exchange of experiences via video calls, among participating providers. 5. Mentions of the programmes’ potential sustainability during meetings with PHCC managers and providers. Continuous communications maintained with the municipal health authorities to promote the application of screening and brief advice.	1. Additional materials were provided for any providers who did not have previous information about the programme. 2. Face-to-face meetings with providers, during which they agreed that no additional tailoring was needed. 3. Reporting each month to PHCCs the number of screenings. 4. Informal exchange of experiences among participating providers. 5. Exploring the option of involving Community Mental Health Services, who could train other centres in the future.

### Questionnaire

The items of the variables *awareness*, *attitude* (*evaluative beliefs*), *subjective norms*, *self-efficacy*, and *intention* were formulated by the research team specifically for the purpose of this study, in order to correspond to the SCALA intervention and the implemented community support. To ensure the content validity, all items were pretested, prior to the start of the intervention, with a group of 10–12 providers in each of the three countries.

### Independent Variable

*Provision of community support* — determined by the assignment to a specific study arm — was coded as a binary variable (1 = community support delivered; in PHCCs from the intervention municipality) or absent (0 = community support not delivered; in PHCCs from the control municipality).

### Mediators

*Awareness* was measured as an index with 10 items, e.g. “I read or heard that alcohol screening and brief advice is simple to deliver”, “I read or heard that alcohol screening and brief advice can help a large number of patients”, “I read or heard about doctors or nurses who were screening and advising many of their patients”, “I was told the number of patients that I am screening and advising” (*yes* = 1, *no* = 0), based on the ten adoption mechanisms and support systems specifically implemented in the project. The score was calculated as the sum of the separate actions, ranging from 0 to 10 (*M* = 7.45, *SD* = 2.53). Cronbach’s alpha could not be calculated, given that the item was measured as an index, rather than scale.

*Attitude* was measured in two ways. First, the shortened version of the Alcohol and Alcohol Problems Perception Questionnaire (hereafter: SAAPPQ domain) measured providers’ attitudes towards delivering brief alcohol advice (Anderson & Clement, 1987) using a seven-point Likert scale (1 = strongly disagree; 7 = strongly agree) developed. The scale includes ten items, for example: “I feel I have the right to ask patients questions about their drinking when necessary”, “I feel I can appropriately advise my patients about drinking and its effects”, or “in general, it is rewarding to work with drinkers”. The score of the SAAPPQ domain was calculated as the average of the ten items (three items were reversed). A higher mean indicated a more positive attitude toward delivery of alcohol measurement and brief advice (*M* = 4.86, *SD* = 0.62; *α* = 0.80).

The second way to measure attitude was with three items measuring evaluative beliefs (hereafter: evaluative beliefs domain), referring to the statement “When I ask my patients about their alcohol consumption…”, for example, “it improves contact with my patients” and “it improves the care of my patients” (1 = completely disagree, 5 = completely agree). A higher mean indicated a more positive attitude toward the delivery of alcohol measurement (*M* = 3.85, *SD* = 0.63, *α* = 0.67).

*Subjective norms* were measured with two items: “My colleagues believe that I should ask my patients how much alcohol they drink” and identically for “my managers” (1 = completely disagree, 5 = completely agree). A higher mean indicated stronger perceived social norms the delivery of alcohol measurement (*M* = 3.04, *SD* = 0.90; *α* = 0.75).

*Self‐efficacy* was measured with four items, referring to the statement “In your daily practice, how difficult or easy do you find…”, for example, “explaining risks to health from different levels of alcohol consumption” or “providing patients with ideas and practical advice on how to cut down”, (1 = very difficult, 5 = very easy). A higher mean indicated a stronger self-efficacy to deliver alcohol measurement and brief advice (*M* = 3.55, *SD* = 0.66; *α* = 0.79).

*Intention* was measured by one statement: “I intend to ask my patients how much alcohol they drink” (1 = completely disagree, 5 = completely agree). A higher mean indicated a higher intention to deliver alcohol measurement (*M* = 4.12, *SD* = 0.69).

### Dependent Variable

*Alcohol measurement rates* were measured as the proportion of patients whose alcohol consumption was measured by the provider (i.e. numerator) out of the total number of consultations delivered by the provider, throughout the 5-month implementation period (i.e. denominator). The alcohol measurements were done using the AUDIT-C questionnaire (Bush et al., 1998) and depending on the patients’ score (below or above the 8-point cutoff) could be followed or not by brief advice and/or referral to treatment. Each alcohol measurement session was recorded by the provider on a separate paper tally sheet, collected afterwards by the research team. The score of the alcohol measurement rates could range from 0 (i.e. none of the consulted patients had their alcohol consumption measured) to 1 (all of the consulted patients had their alcohol consumption measured) (*M* = 0.49, *SD* = 0.12).

### Demographics

*Age* of provider was assessed in years and *gender* of provider was assessed with three answer categories (1 = female; 2 = male; 3 = other).

## Data Analysis

For testing H1, an ANOVA test was used, with alcohol measurement rate as the dependent variable and provision of community support as the independent variable. The country variable was also included as a predictor in the model, to account for possible interaction effects. Age and gender did not differ significantly in the two groups and were, therefore, not included as covariates. The intraclass correlation coefficient (ICC) value of 0.01 at PHCC level indicated that multilevel analyses were not necessary to account for the nested nature of the data.

For testing H2, a path analysis was used, in the programme AMOS 26. The model tested the direct effect of providing community support on providers’ awareness of support. Moreover, a mediation effect was tested on the three socio-cognitive variables (attitude, subjective norms, and self-efficacy), and subsequently on intention, all being measured at the same time, during months 4 and 5 of implementation, i.e. January–February 2020. Error terms between endogenous variables were allowed to correlate freely among themselves. The significance of all indirect effects was assessed using bootstrapping (Kline, 2011). The baseline measurements of attitude, subjective, self-efficacy, and intention were added as predictors of the respective follow-up constructs. It should be mentioned that the relationship between intention and alcohol measurement rates could not be tested in the path model, because of the lack of sufficient behavioural data assessed after the measurement of intention due to the COVID-19 lockdown. Moreover, interactions per country could not be tested because of the limited sample size.

## Results

### Sample Characteristics

For H1, i.e. testing whether the provision of community support leads to increased rates of alcohol measurement sessions delivered by PHC providers, of the 291 providers included in the analysis, 53 were from Colombia, 100 from Mexico, and 138 from Peru. The average age of the respondents was 41.35 years (*SD* = 12.36), with 80.1% being women and 19.9% — men. The professions were as follows: doctor (37.1%), nurse (16.8%), nurse technician (7.9%), psychologist (11.0%), social worker (9.3%), midwife (5.8%), or other professions (12%).

For H2, i.e. testing whether provision of community support leads to (a) increased awareness of support by PHC providers, which consequently leads to a more positive or stronger: (b) attitude; (c) subjective norms; (d) self-efficacy, subsequently resulting in a higher (e) intention to deliver brief alcohol advice*,* of the 139 participants included in the analysis, 47 were from Colombia, 33 from Mexico, and 59 from Peru. The average age of the respondents was 40.15 years (*SD* = 12.12), with 75.5% being women and 15.5% — men. The professions were as follows: doctor (37.4%), nurse (13.7%), nurse technician (14.4%), psychologist (5.8%), social worker (11.5%), midwife (3.6%), or other professions (13.7%). More details regarding the sample characteristics in the control and intervention groups are included in Table 2.

**Table 2:** Descriptive information regarding the age, gender, and profession of the participating providers in the control and intervention groups

	Sample hypothesis 1 (total 291 providers)		Sample hypothesis 2 (total 139 providers)	
	Without community support	With community support	Without community support	With community support
Age	*M* = 42.62, *SD* = 12.50	*M* = 39.82, *SD* = 12.06	*M* = 43.34, *SD* = 12.72	*M* = 37.52, *SD* = 11.01
Gender	Women (80%), men (20%).	Women (79%), men (21%).	Women (74%), men (26%).	Women (76%), men (24%).
Professions	Doctor (35%), nurse (14%), nurse technician (11%), psychologist (14%), social worker (9%), midwife (6%), other (11%).	Doctor (39%), nurse (20%), nurse technician (5%), psychologist (7%), social worker (10%), midwife (6%), other (13%).	Doctor (42%), nurse (11%), nurse technician (6%), psychologists (18%), social worker (7%), midwife (6%), other (10%).	Doctor (34%), nurse (16%), nurse technician (5%), psychologists (12%), social worker (16%), midwife (1%), other (16%).

### Does Community Support Improve Alcohol Measurement Rates?

Provision of community support had a significant small effect on alcohol measurement rates (*F* (1,259) = 4.56, *p* = 0.034, *η*_p_^2^ = 0.018). As hypothesized (H1), providers in the intervention municipal areas where community support was delivered had higher rates of alcohol measurement sessions (*M* = 0.06, *CI* = 0.00 to 1.00), compared to providers in PHCCs where community support was not delivered (*M* = 0.03, *CI* = 0.00 to 0.49). In other words, 6% of the patients consulted by providers who received community support had their alcohol consumption measured, as compared to 3% of the patients consulted by the providers in the control group. A significant effect was also found from the control variable, i.e. country, on the alcohol measurement rates (*F* (2, 259) = 4.11; *p* = 0.017, *η*_p_^2^ = 0.031). Post hoc analyses showed that the alcohol measurement rates were significantly lower in Peru, compared to Mexico (*p* = 0.008, *M*_difference_ = 0.05, *SE* = 0.02), but not between the other country pairs. Moreover, no interaction effect was found between the provision of community support and the country variable, indicating that, in all three countries, the provision of community support led to an increase, albeit small, of the alcohol measurement rates.

### Mechanisms Through Which Community Support Influences Behavioural Intention

For H2, the model fit was evaluated with three indicators: chi-square (should be not significant), RMSEA (should be smaller than 0.05), and CFI (should be higher than 0.95) (Kline, 2011). The model was identified and had an acceptable model fit (*χ*2 (17) = 24.36, *p* = 0.110, RMSEA = 0.06, and CFI = 0.98), allowing to proceed to hypothesis testing. The correlation matrix is included in Table 3.

**Fig. 3:**
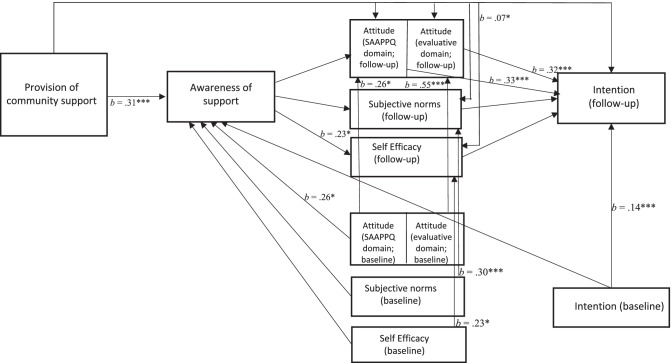
Significant and marginally-significant relationships identified in the path analysis model. Note: *P*-values smaller than 0.001 are indicated by ***, *p*-values smaller than 0.05 are indicated by *

All significant results are shown in Fig. 3, with standardized coefficients. Demographic variables (i.e. age, gender) did not vary significantly between the intervention and control groups and were not included as control variables in the model. The results revealed a positive direct effect of provision of community support on awareness of support (*b* = 0.31, *p* < 0.001). This means that providers in the intervention municipality were more aware of the provided support, compared to providers in the control municipalities. Subsequently, awareness of support had a positive direct effect on providers’ self-efficacy (*b* = 0.23, *p* = 0.010). The higher the awareness of support actions of providers, the higher their self-efficacy to deliver alcohol measurement to their patients. Against expectations, no effect was found of awareness of supportive actions on providers’ attitudes, subjective norms, nor intention.

Both measured domains of attitude (i.e. SAAPPQ and evaluative beliefs) had a positive direct effect on intention (*b* = 0.33, *p* < 0.001 and *b* = 0.32, *p* < 0.001, respectively). The more positive providers’ attitudes towards implementing alcohol measurement, the stronger their intention to deliver the intervention to their patients.

No direct effects were found from provision of community support on intention, nor on any of three mediators: attitude, subjective norms, and self-efficacy. However, results showed an indirect effect of provision of community support on self-efficacy (*b* = 0.07, p = 0.008). This means that community support did influence providers’ self-efficacy to deliver alcohol measurement; however, this effect was fully mediated through the awareness of support.

Controlling for effects of the baseline values of the attitude, subjective norms, and self-efficacy on awareness at follow-up revealed only a positive effect of providers’ baseline attitudes (SAAPPQ domain) on the awareness of support (indicating that a more positive initial attitude led to a higher awareness of support). No other effects of the baseline variables were found on awareness of support. This adds confidence to the direction of the abovementioned found effects, namely that awareness of support influences self-efficacy, rather than the other way around.

## Discussion

This study aimed to assess the effectiveness of community support for bolstering the delivery of an alcohol measurement intervention in a PHC setting. We found a small positive effect of community support on providers’ rates of alcohol measurement delivery, accounting for about 3% more patients receiving alcohol measurements, as compared to the control group. In interpreting this effect, it is important to take into account that the absolute proportion of patients receiving alcohol measurement in the community support group was small in absolute terms (i.e. 6% of the total patients receiving consultations), and the found effect of community support was of low magnitude. However, given the low baseline alcohol measurement rates registered before the launch of the intervention (which were of approximately 1% of the patients receiving consultations), and considering that the provision of community support was stopped prematurely because of the COVID-19 lockdown, these results suggest that the implementation of a full package of community support (e.g. more meetings with providers and implementation of a communication campaign) throughout a longer period of time could in fact lead to stronger effects on the desired behaviour and its socio-cognitive predictors.

Our study is, to the best of our knowledge, the first to show an effect of a relatively complex package of community support on alcohol prevention in primary health care, implemented over the course of several months, in addition to provider training. In contrast to our previous findings (Anderson et al., 2021), where effects of community support were not found, the present analysis focuses on effects at provider level, rather than at PHCC level. This focus at provider level may explain the difference in results, by allowing us to detect differences among implementers at the start of the adoption process. As the diffusion of innovations theory proposes (Rogers, 2010), an intervention will likely first be adopted by fewer persons (i.e. early adopters), before an effect can be observed in the majority of the members of an organization. By analysing the effects at the provider level, implementers and managers can gain valuable insights regarding how to stimulate the adoption and implementation of brief alcohol advice in early phases.

Moreover, our results showed that the delivery of community support helped to increase providers’ self-efficacy to deliver alcohol measurement, but this effect was fully mediated through providers’ awareness of support. This finding gives further underpinning to the observed effect of community support on alcohol measurement rates. This means that in order for a community support to influence providers’ self-efficacy, they need to be aware of this support, in line McGuire’s communication-persuasion model (McGuire, 1989). The community support activities implemented in the current study, before the pause of implementation due to the COVID-19 restrictions, mostly focused on overcoming barriers and promoting facilitators for the delivery of the intervention at the organizational and provider level (as shown in Table 1). Perception of barriers and facilitators are indeed expected to impact self-efficacy beliefs (Craig et al., 2015; Maibach et al., 1991), in line with the results of the current study.

No effects of the community support actions on attitudes, subjective norms, and intention were found. Theoretical explanations for this lack of effects may be that persuasive outcomes such as attitude, subjective norms, and intention generally need a longer time to be changed (Belch & Belch, 2015). Future studies in this area would likely benefit from a longer implementation period to enable the assessment of the effects of community support that may appear over time. Moreover, community support that focuses more explicitly on increasing attitudes, subjective norms, and intentions, for example using targeted communication campaigns or public events (Rice & Atkin, 2012), should be implemented and evaluated.

One of the limitations of this study is that some participants in the control condition may have been exposed to community support, for example in informal discussions during training. Although observations of the training sessions suggest that this has not happened in our project, in future studies, it is important to limit potential contaminations of the control condition by assessing the separate effects of community support without the delivery of training. Moreover, the assessment of the alcohol measurement via paper tally sheets, self-completed by the providers, could have led to less accurate results and/or data loss, as compared to, for example, an automatic electronic registration of the alcohol measurements in an online system. Also, although the ICC did not indicate significant variations at PHCC level, differences in the fidelity of the intervention’s implementation in different PHCCs could have had an impact on the results (Dusenbury et al., 2003). Another limitation is that by agreeing to participate in the study, the providers possibly already had a relatively high intention to deliver the intervention. This may have, on the one hand, led to a ceiling effect that suppressed the potential impact of community support on intention and, on the other hand, made the study less representative for providers who are not inclined to participate in such an intervention. Future studies should explore more in-depth the various motives of providers who are unwilling to deliver alcohol measurement, along with successful recruitment strategies. Finally, it should be noted that, due to the COVID-19 contingencies in participating municipalities, the planned community support could not be fully implemented (for example, the planned communication campaigns were not be implemented). For similar reasons, the sample size is smaller at follow-up due to the abrupt pause in data gathering, which may have been an obstacle in finding more significant effects (Kline, 2011).

An important strength of the study lies in its ecological validity, due to the implementation in a real municipal setting, where the intervention was delivered over several months. This adds confidence to the generalizability of our results, beyond the controlled experimental setting. Moreover, the pre-post quasi-experimental design, with the delivery of community actions as an independent variable, arguably allowed us to detect independent effects of community support, over and above training, increasing the internal validity of the research.

In conclusion, adoption of a health intervention by health care providers may be aided by community support, by directly impacting the rates of alcohol measurement sessions, and by increasing providers’ self-efficacy to deliver this intervention, through increased awareness of support. These results are not only relevant for researchers and practitioners in the field of alcohol control, but also in other health promotion areas.

## Data Availability

According to the project’s data management plan, all quantitative datasets generated in the course of the SCALA study will be made openly available through the UK Data Service upon publication of the results (http://www.data-archive.ac.uk/).

## Annex

**Table 3:** Means, standard deviations and correlations between the variables included in path analysis model

	Mean	SD	Delivery of community support	Awareness of support (follow-up)	Attitude (Evaluative beliefs; baseline)	Attitude (Evaluative beliefs; follow-up)	Attitude (SAAPPQ; baseline)	Attitude (SAAPPQ; follow-up)	Subjective norms (baseline)	Subjective norms (follow-up)	Self-efficacy (baseline)	Self-efficacy (follow-up)	Intention (baseline)	Intention (follow-up)
Delivery of community support	0.56	0.50	1											
Awareness of support (follow-up)	7.45	2.53	0.291^**^	1										
Attitude (Evaluative beliefs — baseline)	3.72	0.63	− 0.109	− 0.058	1									
Attitude (Evaluative beliefs — follow-up)	3.86	0.63	0.002	0.076	0.557^**^	1								
Attitude (SAAPPQ — baseline)	4.78	0.62	− 0.121	0.133	0.556^**^	0.349^**^	1							
Attitude (SAAPPQ — follow-up)	4.86	0.62	0.008	-0.006	0.376^**^	0.433^**^	0.391^**^	1						
Subjective norms (baseline)	3.26	0.96	0.060	0.108	0.125	− 0.100	0.145	0.062	1					
Subjective norms (follow-up)	3.05	0.90	− 0.010	0.048	− 0.151	− 0.250^**^	− 0.004	0.101	0.389^**^	1				
Self-efficacy (baseline)	3.33	0.87	0.098	0.136	0.103	− 0.112	0.123	0.039	0.410^**^	0.355^**^	1			
Self-efficacy (follow-up)	3.55	0.66	− 0.024	0.223^*^	0.130	0.138	0.112	0.286^**^	0.101	0.262^**^	0.240^**^	1		
Intention (baseline)	3.94	0.69	0.085	− 0.017	0.467**	0.280**	0.348**	0.333**	0.316**	0.093	0.188*	.100	1	
Intention (follow-up)	4.12	0.79	0.076	0.140	0.322**	0.535**	0.380**	0.512**	0.047	− 0.121	0.013	.180*	.337**	1

*P*-values smaller than 0.01 are indicated by **, *p*-values smaller than 0.05 are indicated by *
